# Expert Opinion: Place in Therapy of Probiotics in Infertility and Recurrent Implantation Failure

**DOI:** 10.7759/cureus.81067

**Published:** 2025-03-24

**Authors:** Ameet Patki, Sujata Kar, Nayana Patel, Kundan Ingale, Kanthi Bansal, Poornima Durga

**Affiliations:** 1 Gynecology and Obstetrics, Indian Society For Assisted Reproduction (ISAR), Mumbai, IND; 2 Reproductive Medicine, Kar Hospitals, Bhubaneswar, IND; 3 Reproductive Medicine, Akanksha Hospital and Research Institute, Anand, IND; 4 Obstetrics and Gynaecology, Nirmiti Clinic, Pune, IND; 5 Director, Safal Fertility Foundation and Bansal Hospital, Ahmedabad, IND; 6 Infertility, MOM IVF Fertility and Research Centre pvt ltd, Hyderabad, IND

**Keywords:** infertility, in vitro fertilization (ivf), lactobacillus, oral probiotics, recurrent implantation failure

## Abstract

Infertility is a widespread medical condition, affecting a notable percentage of couples globally, with a rising prevalence in India. While assisted reproductive technologies (ART) provide hope, recurrent implantation failure (RIF) continues to challenge approximately one in 10 couples undergoing in-vitro fertilization (IVF). This expert opinion document intends to highlight the promise of probiotics, particularly from Lactobacillus, as a therapeutic approach for improving fertility and treating RIF. In India, on June 29-30, 2024, a physical expert meeting was organized involving 14 specialists from gynecology, obstetrics, and fertility fields. The role of probiotics in reproductive health was discussed in the meeting with an emphasis on infertility and RIF. All experts agree that microbial dysbiosis characterized by an imbalance among the dominant Lactobacillus species is associated with RIF leading to increased inflammation hence poor reproductive outcomes. Probiotics have emerged as possible therapies that restore microbial equilibrium while reducing inflammation and enhancing the intactness of the genital epithelium barrier. All experts have strongly supported the use of Lactobacillus-based oral probiotic supplementation particularly for preventing miscarriages and maintaining pregnancy. All the experts unanimously agreed that oral probiotic supplementation, particularly Lactobacillus-based, should be considered as a potential method to prevent miscarriages and aid in maintaining pregnancy. They recommend oral probiotic use prior to embryo transfer to enhance implantation and pregnancy rates. This expert opinion emphasizes the value of probiotics as a safe and non-invasive approach to treating infertility and RIF. By fostering a balanced microbiome, probiotics may increase the likelihood of successful pregnancies.

## Introduction and background

Infertility is a medical condition that can lead to psychological, physical, mental, spiritual, and medical challenges for the patient. This condition uniquely impacts both the patient and their partner, affecting them as a couple [1]. Infertility is recognized as a condition where a clinical pregnancy is not achieved after 12 months of regular, unprotected sex. It affects 8-12% of couples of reproductive ages globally, with the World Health Organization (WHO) reporting a 17.5% prevalence rate in 2023 [2]. Female infertility, impacting 12.6% of women worldwide, arises from a complex mix of factors, including uterine pathologies, systemic diseases, and age. In India, the infertility rate has significantly increased from 22.4% in 1992-93 to 30.7% in 2015-16 [3]. According to a recent report by the American Council of Reproductive Medicine, infertility is defined as a disease, condition, or status characterized by the inability to achieve a successful pregnancy based on various factors such as medical, sexual, and reproductive history, age, physical findings, diagnostic tests, or a combination of these. It also encompasses the need for medical interventions, including the use of donor gametes or embryos, to achieve a successful pregnancy. For patients having regular, unprotected intercourse without any known reproductive impairments, an evaluation should start after 12 months if the female partner is under 35, and after 6 months if she is 35 or older.

Assisted reproductive technology (ART) offers treatment options for couples struggling to conceive naturally. Despite advances in treatment methods and laboratory technologies, many individuals still face challenges in conceiving through ART. When multiple attempts at in-vitro fertilization (IVF) result in failure, the condition is often referred to as “recurrent implantation failure” (RIF) [4]. RIF is a clinical condition where embryos fail to implant after multiple transfers, affecting about 10% of couples undergoing IVF and embryo transfer [5]. Despite growing research on RIF, there is no universally accepted definition or standard protocol for its diagnosis and treatment. According to a majorly accepted definition, RIF is defined as the inability to achieve a clinical pregnancy after transferring at least four good-quality embryos across a minimum of three fresh or frozen cycles in women under 40 years old [5]. As per the European Society of Human Reproduction and Embryology (ESHRE) good practice recommendations on RIF, RIF refers to a situation where the transfer of presumably viable embryos has repeatedly failed to result in a positive pregnancy test in a particular patient, necessitating further evaluation and/or intervention [4]. The lack of consensus on definitions has hindered progress in predicting and preventing RIF. However, most couples experiencing RIF can achieve pregnancy with clinical intervention [6]. RIF affects 3-4% of women in developed countries and 6-7% in developing countries. Women with RIF are significantly more likely to experience anxiety, depression, stress, and a decreased quality of life, which often leads to sexual dysfunction.

Until the latter half of the 20th century, the endometrial cavity was believed to be sterile. However, advancements in technology and the sequencing of the bacterial 16S rRNA gene revealed that this area has its own distinct microbiome. This microbiome can be classified into two types: Lactobacillus-dominant (LD), where Lactobacillus species make up more than 90% of the population, and non-Lactobacillus-dominant (non-LD), where Lactobacillus species are less abundant [7]. The loss of Lactobacillus dominance in the vaginal microbiota leads to increase microbial diversity and disrupts immune and epithelial balance. This disruption can cause microbial dysbiosis, marked by an abnormal microbiota composition, elevated pro-inflammatory cytokines, and a weakened genital epithelial barrier [8]. Maintaining an "optimal" microbiota in the female reproductive tract is crucial for fertility and pregnancy outcomes. Dysbiosis is linked to negative outcomes such as preterm birth, spontaneous abortion, and infertility [8].

This expert opinion aims to highlight the potential of probiotics as a therapeutic intervention for infertility and RIF. By focusing on the importance of a balanced vaginal and endometrial microbiome, dominated by beneficial Lactobacillus species, the expert opinion underscores how probiotics can restore microbial harmony, reduce inflammation, and enhance the integrity of the genital epithelial barrier. The opinion is grounded in growing scientific evidence that supports the efficacy of probiotics in improving reproductive outcomes, thereby offering a safe and non-invasive approach to addressing microbial dysbiosis. Ultimately, this expert consensus seeks to establish probiotics as a viable option for improving fertility and supporting successful pregnancy outcomes.

## Review

Methods

A physical expert meeting was conducted between June 29 and June 30, 2024, to discuss the use of probiotics in women’s reproductive health, with a particular focus on infertility and RIF. The panel included 14 experts from India with diverse expertise in gynecology, obstetrics, and fertility. The primary objective was to explore the role of probiotics in enhancing women’s reproductive health, including their potential benefits in fertility treatments. Following extensive discussions, key insights from the meeting were compiled into this document. The document was then shared with the experts for review and feedback. Based on their feedback and suggestions, this expert opinion report was prepared and circulated to all experts for final approval.

Factors Affecting Infertility and Recurrent Implantation Failure (RIF)

All the experts agreed that RIF has multifactorial causes, encompassing a range of maternal anatomical factors (Table 1). One of the most critical components influencing RIF is the condition of the endometrium, the lining of the uterus where embryo implantation occurs. Specific endometrial issues, such as a thin endometrium, can create a suboptimal embryo implantation and development environment [6,9]. Additionally, endometrial dysbiosis, characterized by an abnormal endometrial microbiota, further complicates the implantation process. A healthy endometrial microbiome, typically dominated by beneficial Lactobacillus species, is crucial for maintaining immune balance and epithelial integrity. Disruption of this microbiota can lead to increased inflammation and a compromised endometrial lining, significantly affecting implantation success and overall pregnancy outcomes. Therefore, understanding and addressing these endometrial factors is essential in managing and treating RIF effectively. This is supported by evidence from the literature [10-12]. RIF can stem from various factors including maternal anatomic issues (e.g., uterine abnormalities, fibroids), male factors (e.g., severe oligoasthenozoospermia), genetic abnormalities (e.g., aneuploidy in embryos), hormonal or metabolic disorders (e.g., diabetes, thyroid disease), infections, thrombophilias, immunological factors, and psychological or lifestyle factors. Endometrial issues, such as a thin endometrium, and idiopathic causes, where no specific issues are detected despite high-quality embryos, also contribute to RIF [10]. A study by Cela et al. demonstrated that an abnormal endometrial microbiota may impair embryo implantation, leading to RIF in women undergoing IVF [11]. Up to 40% of RIF patients have chronic endometritis, often diagnosed late, linking chronic endometritis and inflammation to pregnancy failure. Evidence indicates a strong connection between endometrial dysbiosis and altered cytokine networks, impacting IVF outcomes [12]. Vaginal dysbiosis, including bacterial vaginosis, may influence endometrial microbiota, affecting implantation. A study by Ichiyama et al. reported that vaginal Lactobacillus levels were associated with RIF. Therefore, treating vaginal infections may enhance endometrial receptivity in RIF patients [13]. This underscores the importance of evaluating uterine microbiota in IVF patients due to its association with inflammation-related changes affecting embryo implantation.

**Table 1:** Experts Comments RIF: recurrent implantation failure; IVF: in-vitro fertilization

Maternal age is a significant independent risk factor for infertility.
Hormonal or metabolic disorders, such as uncontrolled diabetes and thyroid disease, play a crucial role in female infertility.
Sedentary lifestyle and high-fat diet can disrupt the gut microbiome, triggering immune responses that may lead to reproductive health issues and impaired fertility.
An abnormal endometrial microbiota could hinder embryo implantation, potentially contributing to RIF in women undergoing IVF.
Screening for chronic endometritis should be considered in patients with RIF.

The experts further added that unhealthy lifestyles and high-fat diets disrupt the gut microbiome by reducing beneficial bacteria and increasing harmful ones leading to dysbiosis, which further triggers immune system activation, leading to chronic inflammation. In reproductive health, this can contribute to conditions such as polycystic ovary syndrome, endometriosis, and infertility by affecting hormone regulation, menstrual cycle, and implantation resulting in adverse reproductive outcomes such as fertility impairment. Findings from the study by Xiao et al. are similar to this opinion. Sedentariness and high-fat diets, disrupt the gut microbiome and barrier, triggering immune responses and inflammation that can lead to reproductive health issues and impaired fertility [14]. A review by Lakoma et al. also reported that reduced physical activity, increased consumption of hypercaloric, high-glycemic foods with trans fats, and decreased dietary fiber intake negatively affect fertility. Since lifestyle and nutrition are crucial factors in fertility, it is important to raise awareness among couples trying to conceive [15].

The endometrial microbiome, particularly the dominance of Lactobacillus species, plays a critical role in reproductive health. Lactobacillus is known for its protective functions, including the maintenance of a low-pH environment that inhibits the growth of pathogenic bacteria. A healthy endometrial microbiome, characterized by a high abundance of Lactobacillus, is associated with better pregnancy outcomes, especially in ARTs. Disruption of this balance, leading to decreased Lactobacillus dominance, can contribute to chronic endometritis, negatively impacting fertility and pregnancy outcomes [16].

**Figure 1:**
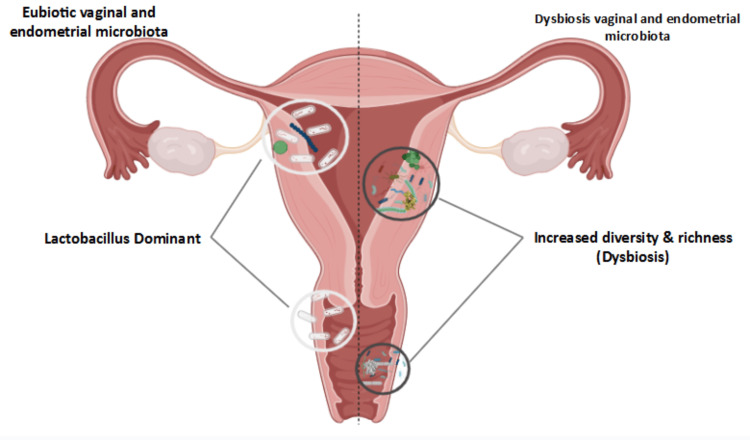
Eubiotic and dysbiotic vaginal and endometrial microbiota This figure was created by the authors, and no copyright permissions were required.

Endometrial dysbiosis refers to an imbalance in the microbial composition of the endometrium, where pathogenic bacteria like *Atopobium*, *Bifidobacterium*, *Chryseobacterium*, *Gardnerella*, *Haemophilus*, *Klebsiella*, *Neisseria*, *Staphylococcus*,* *and *Streptococcus* may dominate over beneficial species like Lactobacillus (Figure 1).

This dysbiotic state is linked to various factors, including antibiotic use, hormonal imbalances, sexual practices, and underlying medical conditions like endometriosis. The consequences of endometrial dysbiosis are significant in the context of reproductive health, particularly regarding RIF. Women with RIF often exhibit an altered endometrial microbiome, which contributes to an inflammatory environment that impairs embryo implantation and reduces the chances of successful pregnancy. Therefore, maintaining a balanced endometrial microbiome is crucial for optimizing reproductive outcomes, especially in ARTs [4,17].

Distinct gut and reproductive microbiota play a significant role in unexplained infertility by influencing various physiological and immunological processes. Dysbiosis can adversely affect reproductive health. In women with unexplained infertility and RIF, distinct profiles of gut and vaginal microbiota have been observed, suggesting a potential link between microbiome health and fertility outcomes [14,18].

Microbiota in the reproductive tract can influence immune responses, which are crucial for successful implantation and pregnancy. Dysbiosis in the reproductive microbiota may lead to inappropriate immune reactions, affecting embryo implantation and increasing the risk of miscarriage [19].

The majority of experts agreed that microbial flora dysbiosis can significantly contribute to female infertility (Table 2). Specifically, an abnormal endometrial microbiota has been identified as a critical risk factor that may impair embryo implantation, leading to RIF in women undergoing IVF. This dysbiosis disrupts the delicate balance needed for a healthy endometrial environment, potentially increasing inflammation and compromising the receptivity of the endometrium to embryo implantation. Addressing and correcting these microbiota imbalances may therefore play a crucial role in improving fertility outcomes for affected individuals. A study by Moreno et al. reported that a dysbiotic endometrial microbiota profile has been linked to unsuccessful IVF outcomes, while a microbiota enriched with Lactobacillus is associated with higher pregnancy rates and live birth outcomes [20]. In line with the consensus, various studies have reported that an abnormal endometrial microbiota has been suggested to impair embryo implantation, leading to RIF and infertility [11,17,21].

**Table 2:** Experts comments RIF: recurrent implantation failure; IVF: in-vitro fertilization

Endometrial dysbiosis should be considered as a potential factor affecting the process of embryo implantation.
Assessing the uterine microbiota should be considered in patients undergoing IVF, given that abnormal microbiota may impair embryo implantation and contribute to RIF.
In the cases of unexplained infertility and RIF, the gut microbiota seems very different.

*Lactobacillus* dominance

Fertility is closely linked to Lactobacillus dominance in the vaginal microbiota. Experts unanimously agreed that a predominance of Lactobacillus species in the vagina correlates with favorable fertility outcomes. Lactobacilli help maintain an acidic environment that supports sperm survival and motility, crucial for successful fertilization. Furthermore, this dominance contributes to a balanced vaginal microbiome that reduces the risk of infections and inflammatory conditions, which can negatively impact fertility. Therefore, promoting and maintaining Lactobacillus dominance is recognized as beneficial for optimizing reproductive health and improving chances of conception. A symbiotic relationship exists between reproductive-age women and their vaginal Lactobacillus species. The vaginal Lactobacilli consume glycogen byproducts produced by estrogenic vaginal epithelial cells and, in return, produce lactic acid, creating a low-pH environment that supports their survival and inhibits opportunistic pathogens [22]. Dysbiosis or imbalance in the endometrial microbiome has been associated with conditions like RIF [11]. A healthy endometrial environment predominantly contains Lactobacillus species, which are essential for maintaining a favorable pH and preventing pathogenic overgrowth [23].

Place of the therapy of Lactobacillus-based probiotics in managing RIF

Probiotics can enhance implantation rates and improve pregnancy outcomes by maintaining a healthy vaginal and endometrial microbiome, particularly through the activity of Lactobacilli, which inhibit pathogenic microbes and reduce infection risks. They also lower inflammation and oxidative stress by producing anti-inflammatory compounds, creating a more favorable environment for embryo implantation. Additionally, probiotics promote a balanced immune response, reducing the chances of immune-related implantation failures, and support endometrial health, which is crucial for successful implantation and overall reproductive health [24-26]. Despite the advances in reproductive medicine, the exact pathophysiology of RIF remains complex and multifactorial. Recent research has highlighted the potential role of the endometrial microbiome and immune modulation in successful implantation, suggesting a promising therapeutic avenue for Lactobacillus-based probiotics [27,28].

The experts highlighted the fact that the endometrial microbiome is crucial for successful implantation as it plays a crucial role in reproductive health and implantation success. All the experts agreed that correcting dysbiosis is a crucial step in the treatment of infertility and RIF. Lactobacillus-based probiotics can help re-establish the balance of the endometrial microbiome, particularly in women with dysbiosis characterized by reduced Lactobacillus and increased pathogenic bacteria [29]. A study has shown that women with a higher proportion of Lactobacillus in their endometrial microbiome have better implantation and pregnancy rates compared to those with dysbiosis [20]. Moreno et al. demonstrated that a higher proportion of Lactobacillus in the endometrial microbiome is associated with better reproductive outcomes, including increased implantation rates [30]. A study by Franasiak et al. showed that patients with RIF had significantly lower levels of Lactobacillus in their endometrial samples compared to fertile women, suggesting that restoring Lactobacillus levels might improve implantation rates [31].

Most experts agree that inflammation in the endometrium can impair implantation. Therefore, screening for inflammatory conditions and prioritizing their treatment, if present, is a recommended approach. Probiotics, particularly Lactobacillus strains, have been shown to have anti-inflammatory properties [32]. These probiotics can reduce the levels of pro-inflammatory cytokines and promote an anti-inflammatory environment [33], which is crucial for successful implantation. By reducing inflammation, Lactobacillus-based probiotics can enhance endometrial receptivity, creating a more conducive environment for embryo implantation [34].

A randomized controlled trial by Reid et al. found that *Lactobacillus rhamnosus* GR-1 and *Lactobacillus reuteri* RC-14 significantly reduced inflammation in women with bacterial vaginosis, indirectly supporting their role in managing inflammation in reproductive health contexts [35]. Literature suggests that probiotics could reduce inflammatory markers in the vaginal microbiota, which might translate to benefits in the endometrial environment as well [33]. A randomized controlled trial by Ghanei et al. showed a significant decrease in inflammatory markers in women who received Lactobacillus supplementation [36].

The majority of experts unanimously agreed that probiotics improve vaginal and gut microbiota, which indirectly supports reproductive health. Probiotics can have a systemic effect on reproductive health through multiple pathways. Probiotics help maintain a healthy vaginal microbiota, which is essential for preventing infections that could interfere with implantation. Dysbiosis of gut microbiota can trigger systemic inflammation and immune function [37], both of which are critical for reproductive health. Lactobacillus-based probiotics support a healthy gut microbiome, which indirectly supports reproductive health by maintaining immune balance and reducing systemic inflammation [38].

A systematic review by Borges et al. highlighted that probiotic supplementation could enhance the vaginal microbiota, reducing the risk of infections that could compromise reproductive outcomes [39]. Furthermore, Saadaoui et al. emphasized the importance of a Lactobacillus-dominated vaginal microbiome in maintaining reproductive health and reducing adverse outcomes like preterm birth and miscarriage [40].

Findings from clinical studies on Lactobacillus supplementation are summarized in Table 3.

**Table 3:** Summary of clinical studies on the use of Lactobacillus supplementation ART: assisted reproductive technology

Authors	Study Design	Study Participants	Intervention	Outcome
Kitaya and Ishikawa (2022) [41]	Prospective pilot study	Women with a history of RIF and non-Lactobacillus-dominant microbiota (NLDM) in vaginal secretions/endometrial fluid. (RIF group, N=117; Non-RIF group, N=55)	Oral enteric coating lactoferrin (700 mg/day for at least 28 days) was administered to eligible RIF patients with NLDM.	-Lactoferrin supplementation improved NLDM in 43.2% of RIF women. - Live birth rate was higher (57.1%) in RIF women with improved microbiota than in those with unimproved microbiota (11.1%).
Thanaboonyawat et al. (2023) [24]	Randomized controlled trial conducted at a fertility clinic from 7 August 2019 to May 2021	340 infertile women undergoing frozen-thaw embryo transfer cycles	One tablet of intravaginal Lactobacilli (*Lactobacillus acidophilus* KS400 bacteria (100 million CFU per tablet)) supplementation prior to embryo transfer.	Lactobacillus supplementation did not improve biochemical or clinical pregnancy rates but significantly reduced the miscarriage rate.
Fernández et al. (2021) [42]	The metataxonomic study	The study involved 58 women aged between 28 and 45 years, comprising 21 with a history of recurrent miscarriages, 23 who had infertility issues despite undergoing at least three rounds of ART, and 14 who were considered fertile controls.	Daily oral administration of *Lactobacillus salivarius* CECT5713 (~9 log_10_ CFU/day) for up to 6 months or until pregnancy.	-56% success rate in women with reproductive failure. -*L. salivarius* CECT5713 significantly improved pregnancy rates and modified key microbiological, biochemical, and immunological parameters in women with reproductive failure.
Schenk et al. (2021) [43]	A prospective, monocentric randomized controlled trial	This study examined probiotics’ influence on infertility in 80 Austrian women with primary or secondary infertility.	Treatment group (n = 40): Received OMNi-BiOTiC® FLORA plus+ probiotic (Four bacterial strains – *L. crispatus* LBV88, *L. rhamnosus* LBV96, *L. gasseri* LBV150N, *L. jensenii* LBV116). 2 g sachet dissolved in 125 ml water daily for 4 weeks, starting on day 20 of the menstrual cycle. Control group: No probiotic supplementation.	-Results indicate that probiotics do not affect vaginal microbiome diversity but help limit *Ureaplasma parvum* growth in treated patients.

Lactobacillus-based probiotics offer a promising therapeutic approach for managing RIF. By restoring a healthy endometrial microbiome, reducing inflammation, and supporting overall reproductive health, these probiotics could enhance the chances of successful implantation and overall fertility outcomes. The evidence from various studies and clinical trials supports their potential role, although more research is needed to fully understand and optimize their use in this context. Table 4 summarizes key expert comments.

**Table 4:** Experts comments

Probiotic supplementation should be recommended, considering its potential to improve ovulation and pregnancy rates.
Oral Lactobacillus supplementation should be considered to potentially prevent miscarriages and support pregnancy maintenance.
A probiotic formulation containing 10 billion CFU, preferably comprising a combination of *Lactobacillus acidophilus* (2 billion), *Lactobacillus rhamnosus* (2 billion), *Lactobacillus reuteri* (2 billion), *Lactobacillus plantarum* (1 billion), *Lactobacillus casei* (1 billion), *Lactobacillus fermentum* (1 billion), *Bifidobacterium bifidum* (1 billion), and Fructo-oligosaccharide (100 mg) is recommended for supplementation 1-2 months prior to embryo transfer to enhance implantation and pregnancy rates in patients undergoing IVF.
Patient education plays a vital role in optimizing reproductive health outcomes. Educating patients on the benefits of a Lactobacillus-dominated microbiome is essential, as current clinical evidence highlights its positive impact on fertility.
Probiotic supplementation should be recommended to enhance implantation and pregnancy rates among patients undergoing IVF.
Understanding the effects of probiotics on the vaginal microbiota in patients with ovarian aging may lead to personalized interventions and better reproductive outcomes.
Probiotic supplementation may create Lactobacillus-dominant microbiota which may improve the chances of successful implantation.
Patient education plays a vital role in optimizing reproductive health outcomes. Educating patients on the benefits of a Lactobacillus-dominated microbiome is essential, as current clinical evidence highlights its positive impact on fertility.

## Conclusions

In conclusion, the current expert opinion from India emphasizes the importance of considering endometrial dysbiosis as a potential factor affecting embryo implantation. It is recommended to assess the uterine microbiota in patients undergoing IVF, as abnormal microbiota may impair embryo implantation and contribute to RIF. Oral probiotic supplementation can induce significant changes in the vaginal microbiota of women undergoing ART, potentially improving their overall bacterial profile. Probiotics help control the growth of non-beneficial bacteria and can prevent or cure dysbiotic vaginal flora. Supplementing with probiotics may create an LD microbiota, which could improve the chances of successful implantation. Therefore, probiotic supplementation is recommended for its potential to improve ovulation and pregnancy rates, support pregnancy maintenance, and enhance implantation and pregnancy rates in patients undergoing IVF.
